# COL-3-Induced Molecular and Ultrastructural Alterations in K562 Cells

**DOI:** 10.3390/jpm12010042

**Published:** 2022-01-04

**Authors:** Mona Fares, Sandra Oerther, Kjell Hultenby, Danica Gubrianska, Ying Zhao, Manuchehr Abedi-Valugerdi, Moustapha Hassan

**Affiliations:** 1 Experimental Cancer Medicine, Division of Biomolecular and Cellular Medicine (BCM), Department of Laboratory Medicine, Novum, Karolinska Institutet, 141 57 Huddinge, Sweden; mona.fares@sll.se (M.F.); sandra.oerther@ki.se (S.O.); danica.gubrianska@gmail.com (D.G.); ying.zhao.1@ki.se (Y.Z.); manuchehr.abedi-valugerdi@ki.se (M.A.-V.); 2 Clinical Research Center and Center for Allogeneic Stem Cell Transplantation, Karolinska University Hospital, 141 86 Huddinge, Sweden; 3 Department of Laboratory Medicine, Karolinska Institutet and Karolinska University Hospital, 141 57 Huddinge, Sweden; kjell.hultenby@ki.se

**Keywords:** K562 cells, paraptosis, programmed necrosis, COL-3, m-calpain

## Abstract

Tetracycline-3 (4-dedimethylamino sancycline, COL-3) is a non-antibiotic tetracycline derivative. COL-3 exerts potent anti-metalloproteinase activity and its antitumor effects have been reported both in vitro and in vivo. In this study, we investigated the mechanisms of COL-3-induced cytotoxicity in a chronic myeloid leukemia cell line, K562, characterized by the BCR–ABL fusion protein. COL-3 induced K562 cell death in a concentration-dependent manner with an IC50 of 10.8 µg/mL and exhibited features of both apoptosis and necrosis. However, flow cytometry analysis revealed that necrotic cells dominated over the early and late apoptotic cells upon treatment with COL-3. Transmission electron microscopy analysis in combination with Western blotting (WB) analysis revealed early mitochondrial swelling accompanied by the early release of cytochrome c and truncated apoptosis inducing factor (tAIF). In addition, ultrastructural changes were detected in the endoplasmic reticulum (ER). COL-3 affected the levels of glucose-regulated protein-94 (GRP94) and resulted in m-calpain activation. DNA double strand breaks as a signature for DNA damage was also confirmed using an antibody against γH2AX. WB analyses did not demonstrate caspase activation, while Bcl-xL protein remained unaffected. In conclusion, COL-3-induced cell death involves DNA damage as well as mitochondrial and ER perturbation with features of paraptosis and programmed necrosis.

## 1. Introduction

A balance between cell proliferation and cell death is mandatory for normal development and tissue homeostasis and disturbing this balance may contribute to the development of malignancy [1]. Cell death may be divided into apoptosis and necrosis based on the morphological and biochemical features of dying cells [2]. Apoptosis is a highly conserved programmed cell death controlled by caspases, a family of cysteine proteases [3]. Depending on the initiating factor(s), apoptosis is mediated by extrinsic or intrinsic pathways [2]. While the extrinsic pathways are initiated by extracellular stressors through trans-membrane receptors, the intrinsic pathways are associated with intracellular stressors that cause DNA damage, oxidative stress or perturbation of intracellular organelles [2,4]. In contrast to apoptosis, necrosis is considered to be an uncontrolled cell death characterized by a loss of membrane integrity and dilatation of cellular organelles or as a programmed event that is mediated by receptor-interacting protein kinases (RIP)-1 and RIP-3 [5].

Tetracyclines (TCs) are heterogeneous broad-spectrum antibiotics. The chemical and physical properties of TCs facilitate their interaction with a variety of bacterial or mammalian targets including ribosomes, mitochondria, DNA, proteins, lipid molecules and cell membrane receptors [6,7]. The TC, 4-dedimethylamino sancycline (COL-3), is a chemically modified TC with no 4-dimethylamino, 6-hydroxyl and 6-methyl groups [8]. COL-3 has been reported to inhibit the proliferation and migration of several types of cancer cells and its anticancer effects have been reported to be associated with the early disruption of the mitochondrial membrane potential (Δψm) [9,10].

Based on the anti-proliferative effect of COL-3 on solid tumor cells, we hypothesized that this compound may also induce similar effects on leukemia cells. This hypothesis was supported by our earlier findings that COL-3 induced cell death in human acute myeloid leukemia (AML) HL-60 cells in a caspase-dependent manner [11].

COL-3-induced cell death in AML cells raised another question as to whether COL-3 has a similar anti-cancer effect against chronic myeloid leukemia (CML) cells. The answer to this question is of great importance because AML and CML exhibit clear biological and genetic differences [12]. For instance, BCR–ABL, a constitutively active tyrosine kinase is exclusively present in CML cells and confers resistance to conventional chemotherapy [13]. The present study was designed to investigate the cell death-inducing effects of COL-3 on a K562 cell line derived from a CML patient with blast crisis expressing both BCR–ABL and the anti-apoptotic molecule, Bcl-xL, but not the tumor suppressive molecule, p53 [14].

## 2. Materials and Methods

### 2.1. Cells Lines and Cell Culture

The K562 cell line was from the American Type Culture Collection (ATCC); the HL-60, Jurkat and KG1a cell lines were from DSMZ (Leibniz Institute, DSMZ-German Collection of Microorganisms and Cell Cultures GmbH, Braunschweig, Germany). Cells were cultured in an RPMI 1640 medium supplemented with 10% heat-inactivated FBS at 37 °C in a 95% humidified atmosphere containing 5% CO_2_. All experiments were performed in exponentially growing cells with passage numbers <10, and the cells were tested routinely for mycoplasma contamination using a commercially available mycoplasma detection kit (Lonza, Basel, Switzerland).

### 2.2. Cell Treatment

COL-3 was generously provided by Galderma (Sophia Antipolis; France). Cells were treated with COL-3 at final concentrations of 2.5, 5, 10, 20 and 50 µg/mL for different time periods (1 min to 72 h). DMSO (0.1%) and etoposide (6 or 40 µg/mL) were used as solvent and positive controls, respectively. To assess the effects of pancaspase inhibitor Z–VAD–fmk/RIPK1 inhibitor nescrostatin-1, cells were first treated with Z–VAD–fmk/nescrostatin-1 for 1 h, followed by co-incubation with COL-3.

### 2.3. Measurement of Cytotoxicity

Cytotoxicity was assessed using a resazurin viability assay. Ten thousand cells per well were seeded on 96-well black microplates and incubated in triplicates with COL-3 at final concentrations of 2.5–50 µg/mL for 24 h at 37 °C. After incubation, resazurin was added to each well and the plates were incubated for 2 h at 37 °C. Fluorescence was read using FLUOstar Optima (BMG Labtech, Ortenberg, Germany) at a wavelength of 590 nm. The 50% inhibitory concentration (IC50) was calculated as the drug concentration inducing a 50% reduction of the cell viability. The data were fitted into a nonlinear regression model using GraphPad Prism 6 (GraphPad Software Inc. San Diego, CA, USA).

### 2.4. Cell Death Assessment

Cells with features of apoptosis were characterized by condensed chromatin and/or fragmented nuclei and features of necrosis as characterized by increased cell size, loss of cellular architectures and reduced cellular basophilia. Apoptosis or necrosis was expressed as the percentage of the number of affected cells/400 counted cells per slide.

Cell death was also assessed using PI and annexin V staining. The apoptotic/necrotic cells were distinguished by a FACScan flow cytometer (BD) and analyzed by Flowing Software, version 2.5.1 (Cell Imaging Core, Turku Centre for Biotechnology, Turku, Finland).

### 2.5. Transmission Electron Microscopy (TEM)

COL-3-treated K562 cells were first fixed in a phosphate buffer containing 2.5% glutaraldehyde and kept overnight at 4 °C. After fixation, the cells were rinsed and postfixed in 2% osmium tetroxide, dehydrated by ethanol and acetone and embedded in resin LX-112. Ultrathin sections (approximately 40–50 nm) were cut using a Leica EM UC 6. Sections were contrasted with uranyl acetate followed by lead citrate and examined in a Tecnai 12 Spirit Bio TWIN transmission electron microscope (Fei Company, Eindhoven, The Netherlands) at 100 kV. Digital images were taken with a Veleta camera (Olympus Soft Imaging Solutions mbH, Münster, Germany).

### 2.6. Protein Analysis Using Western Blotting

In studies with whole cell lysates, cells were lysed with a lysis buffer containing protease inhibitors. The lysates were then centrifuged, and the supernatants were collected. To assess poly (ADP-Ribose) polymerase (PARP), the cells were incubated in a PARP extraction buffer and prepared for Western blotting (WB). For subcellular fractionation, cells were incubated in a digitonin cytosolic buffer, then centrifuged and the supernatant collected. Pellets were either re-suspended in PBS containing protease inhibitors, or further separated into cytosolic and nuclear fractions using NE–PER^®^ Nuclear and Cytoplasmic Extraction Reagents (Pierce, Rockford, IL, USA) according to the manufacturer’s instructions.

Protein concentrations were determined using the Pierce^®^ BCA assay kit (Pierce, Rockford, IL, USA) according to the manufacturer’s instructions. For WB, the protein samples were separated by SDS–PAGE and transferred to poly(vinylidene) fluoride (PVDF) or nitrocellulose membranes. The following primary antibodies were used to detect the desired proteins: rabbit antibodies against caspase-3, -9, γH2AX (20E3), glucose-regulated protein 94 (Grp94) (Cell Signaling Technology, Danvers, MA, USA); rabbit antibodies against caspase-8, Bcl-xL apoptosis-inducing factor (AIF) (BD); rabbit antibodies against actin (Sigma); rabbit antibodies against m-calpain (Millipore Corporation); mouse antibodies against cytochrome c (BD); mouse antibodies against PARP (C-2-10) (Oncogene Research Products); and mouse antibodies against AIF (B-9) (Molecular Probes, Carlsbad, CA, USA). Anti-rabbit or anti-mouse antibodies conjugated with either peroxidase (Amersham Pharmacia Biotech AB, Uppsala, Sweden) or IRDye^®^ 800CW or IRDye^®^ 680CW (LI–COR, Lincoln, NE, USA) were used as the secondary antibodies. The proteins were visualized using the ODYSSEY imaging system (LI–COR, Lincoln, NE, USA). The plots were cropped and thereafter the contrast and brightness were adjusted using the software/program Image J.

### 2.7. Intracellular Reactive Oxygen Species Assay

Changes in the intracellular reactive oxygen species (ROS) levels were measured using the Intracellular ROS Assay Kit (OxiSelect^TM^, Cell Biolabs, San Diego, CA, USA) in accordance with the manufacturer’s instructions. The changes in dichlorofluorescein (DCF) fluorescence intensity were measured at wavelengths 485/520 nm using FLUOstar Optima.

### 2.8. Immunocytochemistry (ICC)

COL-3-treated K562 cells were fixed with paraformaldehyde (PFA), permeabilized with Triton X-100 and blocked for internal peroxidase activity. Thereafter, the cells were incubated with rabbit anti-γH2AX followed by an anti-rabbit HRP-labeled Polymer (Dako A/S, Glostrup, Denmark). Color development was assessed using the chromogen, 3,3′Diaminobenzidine (DAB) and hematoxylin.

### 2.9. Statistical Analysis

Experiments were performed independently three times and the results are expressed as the mean ± SD where appropriate. The statistical significance of differences was determined by the two-factor analysis of variance with replication followed by Tukey’s post hoc analysis. A *p*-value of <0.05 was statistically significant.

## 3. Results

### 3.1. Effects of COL-3 on the Viability of K562 Cells

**Figure 1:**
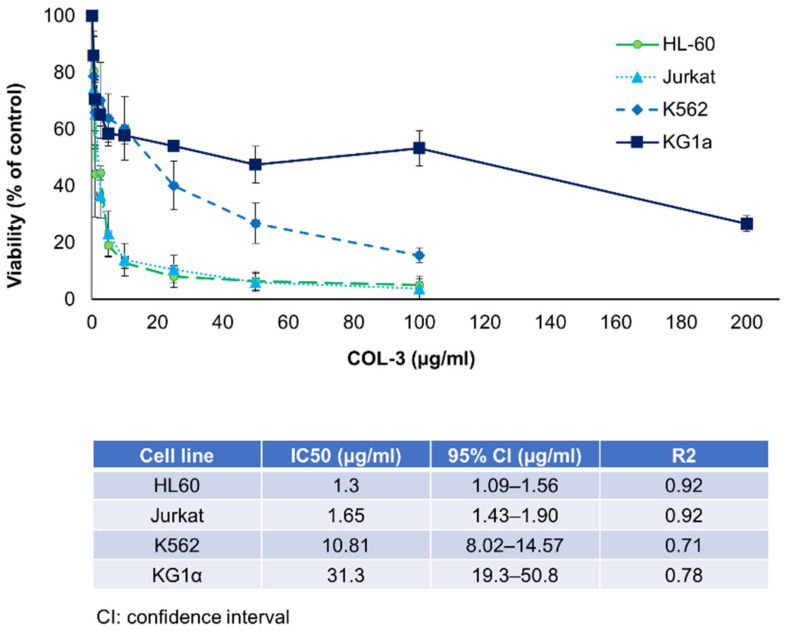
The effect of COL-3 on the viability of human leukemic cell lines. The leukemic cell lines were incubated with COL-3 in concentrations within a range of 0.5–200 µg/mL. Cells incubated in complete medium served as controls. Cell viability was studied using resazurin fluorescence assay and expressed as a percentage of the control. In all experiments, the DMSO final concentration did not exceed 0.2% and showed no cytotoxic effects. Results are expressed as mean ± SD of three independent experiments.

The anti-proliferative effects of COL-3 were first evaluated on four leukemia cell lines. The IC50 for the HL-60, Jurkat, K562 and KG1a cell lines were found to be 1.30, 1.65, 10.81 and 31.30 (µg/mL), respectively (Figure 1). In a mouse leukemia cell line, C1498, the IC50 of COL-3 was determined to be 37 µg/mL (Figure S1).

### 3.2. Characteristics of COL-3-Induced Cell Death in K562 Cells

**Figure 2:**
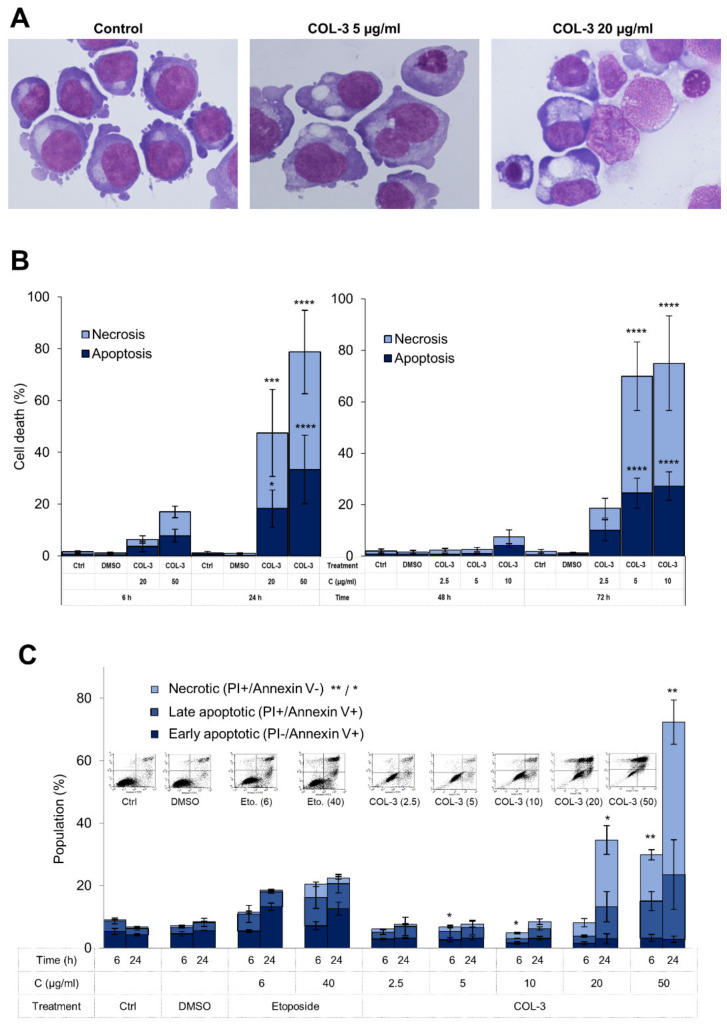
COL-3-induced cytotoxicity in K562 cells. K562 cells were treated with COL-3 (C, 2.5–50 µg/mL) for 72 h. May–Grünwald-stained cells with morphological features of apoptosis (chromatin condensation) and necrosis (increased cell size and cytoplasmic vacuolization) (**A**) were counted as a percentage of 400 cells per slide (**B**). (**C**) The cells were also stained with Annexin V and PI and subsequently analyzed by flow cytometry. Necrotic cells were designated as PI+/Annexin V− cells, whereas PI−/Annexin V+ cells and PI+/Annexin V+ cells were considered as early and late apoptotic cells, respectively. In (**B**,**C**), cells treated with etoposide (E, 40 µg/mL) were used as the positive controls, while vehicle DMSO (D)-treated and/or untreated cells were employed as normal controls. Representative flow cytometry images for treatments after 24 h are shown in the upper part of Figure 2C. Results are expressed as the mean ± SD of three independent experiments. *: *p* < 0.05; **: *p* < 0.01; ***: *p* < 0.001, and ****: *p* < 0.0001.

To evaluate the possible cytotoxic effect of COL-3 on K562 cells, we first performed a morphological analysis of these cells upon exposure to COL-3 using light microscopy. As shown in Figure 2A–C, in a time- and concentration-dependent manner (Figure 2B), COL-3 induced morphological alterations which were representative of both apoptosis (chromatin condensation) and necrosis (increased cell size and cytoplasmic vacuolization) (Figure 2A).

Next, we performed flow cytometry analysis to determine whether COL-3 promotes cell death in K562 cells and if so, what the pattern of COL-3-induced cell death is. As shown in Figure 2C, after 6 h and 24 h of COL-3 exposure, cells showed a dominance of necrotic (PI+/Annexin V−) cells over the late apoptotic (PI+/Annexin V+) cells. A smaller population of treated cells exhibited an early apoptotic (PI−/Annexin V+) pattern (Figure 2C).

### 3.3. Induction of Ultrastructural Changes in K562 Cells by COL-3

**Figure 3:**
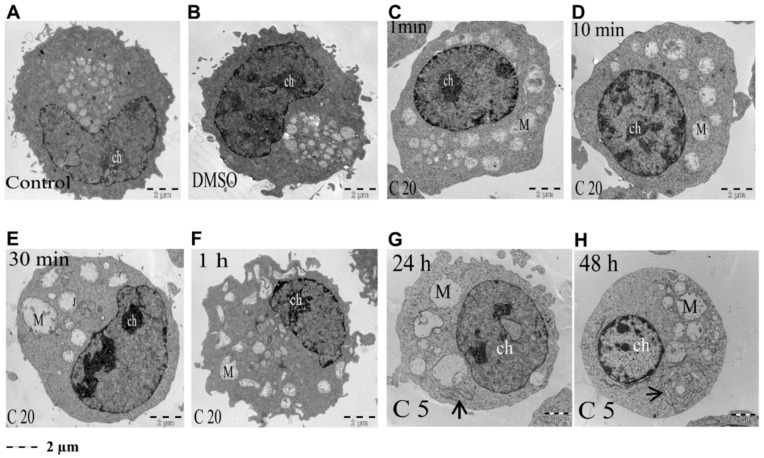
COL-3-induced morphological changes assessed with TEM (low magnification). K562 control cells were left untreated (**A**) or were treated with vehicle DMSO (**B**) or with 20 µg/mL COL-3 for 1 min, 10 min, 30 min and 1 h (**C**–**F**, respectively) and 5 µg/mL COL-3 for 24 h and 48 h (**G**,**H**, respectively). Thereafter, ultrastructural morphology was assessed by TEM. M = mitochondria; ch = chromatin; arrows indicate ER.

Our results showing that COL-3 induced cell death in 562 cells led us to further characterize the sub-cellular alterations caused by COL-3 employing TEM. The untreated and DMSO-treated control K562 cells exhibited pleomorphic blebs over the cell surface (Figure 3A,B). In addition, an eccentric kidney-shaped nucleus and a cytoplasm containing multiple mitochondria (elongated, round or branched) were also observed in both controls (Figure 3A,B).

**Figure 4:**
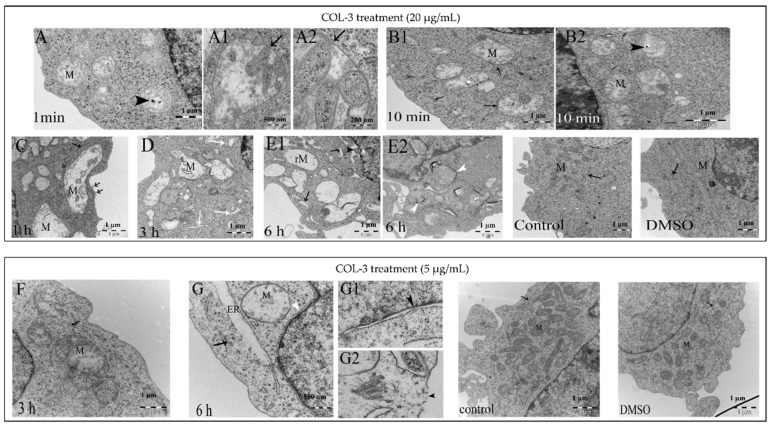
COL-3-induced ultrastructural changes (high magnification). K562 cells were treated with COL-3 (20 µg/mL) for 6 h (**A**–**E**) or (5 µg/mL) for 72 h (**F**,**G**). K562 control cells were untreated and/or treated with DMSO. Ultrastructural morphology was assessed with TEM. The following changes were visible in COL-3-treated (20 µg/mL) cells: (**A**): After 1 min, swollen mitochondria (M) with disintegrated cristae and electron dense structure (black arrowhead). (**A1**,**A2**): higher magnification (500 nm) revealed intact mitochondrial membranes (black arrows). (**B1**,**B2**): After 10 min, the ER are approximated to mitochondria and devoid of ribosomes (closed head arrow). (**C**): After 1 h, early disruption of the plasma membrane (open head arrows). (**D**): After 3 h, ER dilatation with no ribosomes (white arrows). (**E1**,**E2**): After 6 h, ER and perinuclear cisternae dilatation (arrows and black arrowhead); ruptured mitochondria (rM) are enclosed in autophagosomes (white arrowhead). Control cells (DMSO-treated and untreated) show elongated mitochondria (M) with normal cristae and short segments of rough endoplasmic reticulum (closed head arrow). The following changes were visible in COL-3-treated (5 µg/mL) cells (**F**): After 3 h, swollen mitochondria with displaced cristae (M) and mildly dilated ER (closed head arrow). (**G**): After 6 h, extensive mitochondrial swelling (M) with ruptured outer membranes, ER dilatation with disintegrated membranes connected to the dilated perinuclear cisternae and no ribosomes (white arrowhead). (**G1**,**G2**): Higher magnification (500 nm): Damaged nuclear and plasma membranes and disintegrated nuclear pore complex (black arrowheads). Controls: DMSO-treated and untreated cells after 72 h.

Treatment of K562 cells with a high concentration of COL-3 (20 µg/mL) induced rapid (within 1 min) mitochondrial (M) swelling (Figure 4A) along with cristae that had disintegrated into vesicular and tubular structures (Figure 4A1,A2). Upon prolongation of the treatment time (10 min, 30 min, 1 h, 3 h and 6 h), this mitochondrial swelling/rupture became more pronounced and, in addition, a progressive dilatation of the ER with detached ribosomes and chromatin condensation was observed (Figure 3D–F and Figure 4B1,B2,C,D,E1,E2).

COL-3 at a low concentration (5 µg/mL) also exerted similar subcellular changes in K562 cells, although a longer incubation period was required (Figure 3G,H and Figure 4F,G,G1,G2).

### 3.4. The Role of Mitochondrial Mediators of Apoptosis in COL-3-Induced Cell Death in K562 Cells

**Figure 5:**
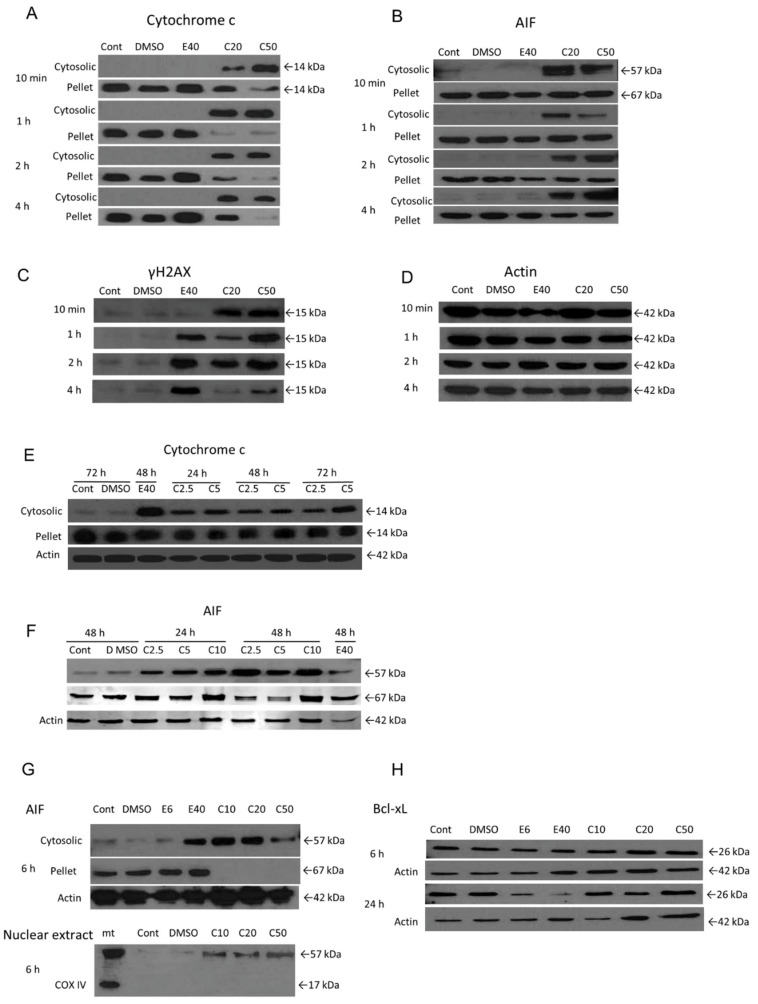
The role of mitochondria and DNA damage in COL-3-induced cell death. Cells were treated with COL-3 (C, 2.5–50 µg/mL) for 72 h and processed for subcellular fractionation and Western blotting as described in the Materials and Methods section. Controls included etoposide-treated cells (positive control) (E, 6 or 40 µg/mL), DMSO treated (vehicle control) and untreated cells. Cytosolic translocation of cytochrome c (**A**,**E**) and AIF (**B**,**F**); protein expression of γH2Ax (**C**); AIF nuclear translocation (**G**); protein expression of Bcl-xL (**H**). (**D**) represents the house-keeping protein (Actin, 42 kDa) associated with (**A**–**C**). COX IV = cytochrome c oxidase complex IV subunit II; mt = mitochondrial-rich fraction.

To understand the COL-3-induced ultrastructural alterations in mitochondria described above, we moved on to investigate the molecular changes involved in these alterations. We first determined whether COL-3-induced mitochondrial swelling results in the release of cyt c into the cytoplasm. WB analysis showed that high concentrations of COL-3 (20 and 50 µg/mL) induced a cytosolic translocation of cyt c as early as 10 min (Figure 5A). At concentrations of 2.5 and 5 µg/mL, the cyt c translocation occurred after 24 h (Figure 5E).

Next, we performed an experiment to determine whether the mitochondrial-associated protein AIF also plays a role in COL-3-induced cell death. Like Cyt-c, AIF is normally located in the mitochondrial intermembrane space of a variety of cancer cell lines. Upon induction of apoptosis, AIF translocates through the outer mitochondrial membrane to the cytosol and thereafter, to the nucleus. Translocation of AIF to the nucleus induces nuclear chromatin condensation, as well as DNA fragmentation [15]. In our experimental set up, the release of mitochondrial AIF (cytoplasmic tAIF, 57 kDa) could also be detected at 10 min post-treatment with 20 and 50 µg/mL of COL-3 (Figure 5B) or after 24 h at COL-3 concentrations of 2.5 and 5 µg/mL (Figure 5F). Total AIF (67 kDa) could be detected in the pellet fractions for up to 4 h (Figure 5B) and then disappeared after 6 h (Figure 5G) of treatment with 20 and 50 µg/mL COL-3.

Accordingly, the loss of mitochondrial membrane potential (ΔΨm) was observed in COL-3 treated K562 cells (Figure S2). The decrease in ΔΨm progressed in a concentration- and time-dependent manner, which was detected as early as 2 h after COL-3 treatment.

Unlike cyt c and AIF, there were no changes in the protein levels or cleavage of the Bcl-xL molecule even after 24 h of exposure to COL-3 (Figure 5H). Thus, COL-3-induced mitochondrial swelling can result in the release of the cell death-inducing proteins, cyt c and AIF, from these organelles.

### 3.5. COL-3 Induced DNA Damage in K562 Cells

DNA damage has been known to be one of the main routes that leads to apoptosis [16]. Thus, we performed WB and immunocytochemistry assays to determine whether COL-3 promotes DNA damage in K562 cells. As demonstrated in Figure 5C and Figure S3, COL-3 at concentrations of 20 and 50 µg/mL caused progressive DNA double strand breaks which were detected after 10 min and intensified by 2 h. COL-3 (<10 µg/mL) required a longer time to induce DNA damage (data not shown).

### 3.6. Relationship between COL-3-Induced Dilatation and the Development of Cell Death

**Figure 6:**
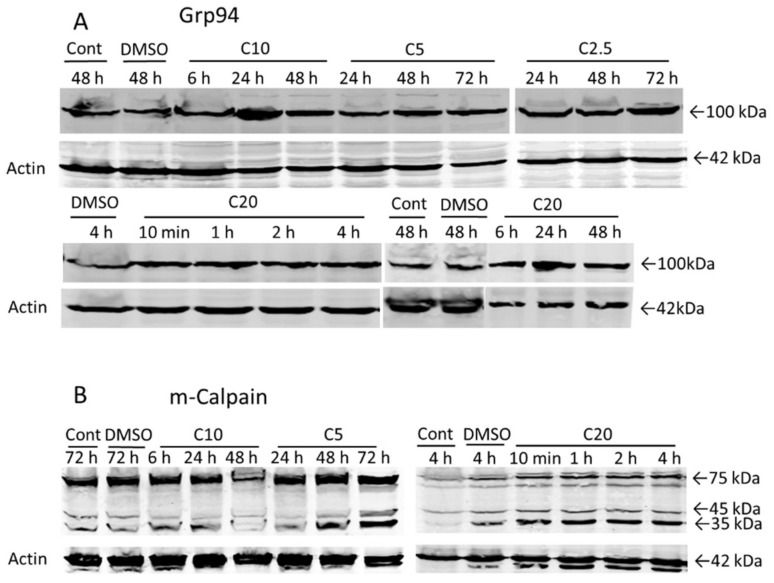
The role of ER damage in COL-3-induced cell death. Cells were treated with COL-3 (C, 2.5–20 µg/mL) for 72 h and processed for Western blotting. Controls included DMSO (D)-treated (vehicle control) and untreated cells. Protein expression of Grp94 (**A**) and activation of m-calpain (**B**).

It is well established that specific ER stress signaling pathways (known as the unfolded protein response UPR), are required for the maintenance of ER homeostasis. The UPR is triggered when the ER protein folding capacity is overwhelmed by cellular demand and the UPR initially aims to restore ER homeostasis and normal cellular functions. However, if the restoration of ER homeostasis fails, the UPR triggers cell death [17]. To investigate the role of ER dilatation in COL-3-induced cell death, we performed WB analysis on the protein expression pattern of Grp94 and m-calpain, the two ER proteins that play a crucial role in cell death caused by ER stress [18]. Grp94-protein expression increased after COL-3 treatment (Figure 6A). The activation of m-calpain was detected by the presence of two cleavage products of 35 and 45 kDa. COL-3 (20 µg/mL) induced m-calpain cleavage within 10 min and lower concentrations of COL-3 induced this cleavage at later points (Figure 6B).

### 3.7. The Role of Caspase Activation and ROS Formation in COL-3 Induced Cell Death

The contribution of caspases and ROS formation in COL-3-induced cell death was also investigated. WB analysis did not support the activation of the crucial caspases (9 and 3) for initiating the apoptotic pathway (Figure S4). Neither the 85 KDa PARP-1 cleavage fragment (apoptosis), nor the 50 KDa fragment (necrosis) were detected (data not shown). Furthermore, pre-treatment with the pan-caspase inhibitor, Z–VAD–FMK, did not affect COL-3-induced cytotoxicity as assessed by morphology (Figure S5A). Necrostatin-1 is a well characterized inhibitor of receptor-interacting serine/threonine-protein kinase 1 (RIPK1), which is able to block necrotic cell death. Pre-treatment of K562 cells with Necrostatin-1 showed less cell death upon COL-3 treatment (Figure S5B). Intracellular ROS levels, as assessed by the DCFH–DA probe showed no change following treatment with COL-3 (20 µg/mL) (Figure S6).

### 3.8. Effect of Extracellular Ion Concentrations on COL-3-Induced Toxicity

To further investigate the role of extracellular ions in COL-3-induced ultrastructural changes, cells were treated with COL-3 in four different buffers with different extracellular ion concentrations (Figure S7). In the presence of high Na^+^ and low K^+^, along with the absence of Ca^2+^ and Mg^2+^ (DPBS) in the incubation buffer, the mitochondrial swelling (black arrow) became more prominent. Replacing the Na^+^ with K^+^ (KPBS) did not induce complete mitochondrial recovery.

## 4. Discussion

Our previous study indicated that the TC analogues, doxycycline, minocycline and COL-3 were able to induce caspase-dependent apoptosis in HL-60 leukemia cells and revealed that COL-3 was superior to the other compounds [11]. In the present study, we assessed the anti-proliferative effect of COL-3 on K562 leukemia cells that represent CML blast crisis cells [19,20]. Our results showed that leukemia cells are also susceptible to COL-3-induced cytotoxicity at moderate concentrations during the early stages of differentiation.

Light microscopy analysis demonstrated that COL-3 treatment caused cytoplasmic vacuolization and chromatin condensation, which represent the morphological characteristics of both apoptotic and necrotic cells. Furthermore, flow cytometry analysis revealed that among COL-3-treated K562 cells, necrotic cells represented the largest population followed by late apoptotic cells. Interestingly, the early apoptotic cell population remained small. These findings, in combination with a lack of caspase activation, as shown by WB protein analysis, and the inefficacy of the pan-caspase inhibitor, Z–VAD–FMK, on COL-3-treated K562 cells, imply that necrosis is dominant over apoptosis in COL-3-induced cell death in K562 cells.

Necrotic cells are defined as cells that contain dilated cytoplasmic organelles (e.g., mitochondria, ER and Golgi apparatus) along with a moderately condensed chromatin [3]. Consistent with this definition, TEM analysis demonstrated that in a concentration- and time-dependent manner, COL-3 induced extensive mitochondrial swelling, ER dilatation, chromatin condensation and ribosomal detachment in K562 cells. These observations strongly support the above-mentioned hypothesis that COL-3 mainly activates necrotic pathways in K562 cells.

A novel and interesting observation was that COL-3 induced the detachment of ribosomes from the ER. Since the synthesis of secretory proteins takes place in membrane bound ribosomes [21], it is possible that the detachment of these organelles impairs the synthesis of secretory proteins in K562 cells. This is consistent with the finding that COL-3 directly targets human ribosomes and thereby exerts anti-proliferating effects on K562 cells [22].

The earliest molecular events associated with COL-3-induced cell death were the release of the mitochondrial mediators, cyt c and tAIF. These observations, in combination with mitochondrial swelling/rupture, strongly suggest that the loss of the mitochondrial membrane potential (MPT, Δψm) and the opening of the permeability transition pore (PTP) are involved in the process of cell death caused by COL-3. In the PTP, the interaction between adenine nucleotides, voltage-dependent anion channels and Bax (Bak) [23] results in widening of the pores, a phenomenon that facilitates the passage of larger molecules such as cyt c and tAIF. This process is naturally inhibited by the Bcl-xL molecule [24]. Our finding that COL-3 treatment did not affect Bcl-xL expression or cleavage implies that the opening of the mitochondrial PTP occurs independently of Bcl-xL expression.

Other factors that may contribute to the loss of MPT are oxidative stress and increased mitochondrial Ca^2+^ [25]. Since treatment with COL-3 did not induce ROS formation in K562 cells, the contribution of this factor to the MPT can be ruled out.

Several findings in this study suggest that mitochondrial calcium overload plays a pivotal role in the loss of MPT in COL-3-induced cell death. First, m-calpain cleavage occurs early and two fragments of 35 and 45 kDa were detected. These fragments most likely originate from the 76 kDa mitochondrial m-calpain, which generates a 40 kDa fragment upon activation [26]. Second, the presence of multiple contact sites between the ER and mitochondria, as detected by TEM analysis, may indicate increased mitochondrial Ca^2+^ uptake [27]. Third, the electron dense structures observed in the mitochondrial matrix may correspond to calcium precipitates, as similar structures have been found as a result of calcium accumulation in the mitochondria [28].

Ca^2+^ may have an extracellular or intracellular origin. The cation ionophoric properties of COL-3 [6] may facilitate extracellular calcium diffusion through the cell membrane. Our results showing that the depletion of extracellular Ca^2+^ did not reverse the ultrastructural mitochondrial changes induced by COL-3 suggest that Ca^2+^ might be mobilized from a storage site inside the cell. This explanation is in line with our observation that ultrastructural changes in the ER mainly occurred near mitochondria.

ER dilatation and Grp94 protein expression upregulation were reported to be associated with ER Ca^2+^ release [29,30]. Rapid ER dilatation and an increase in Grp94 protein expression after exposure to COL-3 suggest that COL-3 treatment induces ER Ca^2+^ mobilization.

Since the COL-3-induced MPT state is apparently independent of Bcl-xL, ROS formation or extracellular Ca^2+^ depletion, we assessed the role of Na^+^ and K^+^ ions in COL-3-induced effects on mitochondria. We found that mitochondrial swelling increased when the medium contained high Na^+^ and low K^+^ ion concentrations. However, increasing the K^+^ concentration in the medium did not reverse COL-3-induced mitochondrial damage.

We observed that COL-3-induced DNA damage was proven by γH2AX positivity. However, as the DNA damage occurred early, it is possible that COL-3 affects the DNA directly. This is consistent with the reported in vitro interactions between TCs and DNA [7]. DNA damage is associated with increased PARP-1 activity [31]. However, in our study, the PARP-1 protein remained intact until 24 h after COL-3 treatment. DNA double-strand breaks (DSBs) activate apoptosis signals mediated by ataxia telangiectasia mutated (ATM) kinase and p53 proteins [32]. Other reports [14,19] have demonstrated that K562 cells are devoid of both wild type and mutant p53. Thus, it is likely that COL-3 induces cell death in a p53-independent manner. This likelihood requires further investigations in which COL-3-induced cell death is examined in other CML blast crisis cell lines as well as in CML cells that express active p53. We are in the process of conducting research that involves these types of investigations.

## 5. Conclusions

In summary, our results demonstrated that COL-3 targeted the mitochondria and nuclear DNA in K562 cells. We observed that, at concentrations above the IC50, both mitochondria and DNA were affected, while at lower COL-3 concentrations the mitochondria appeared to be the primary target. As summarized in Supplementary Table S1, COL-3-induced cell death in K562 cells primarily had the features of programmed necrosis [33]. Thus, our findings concerning COL-3-induced cell-death can be added to the reported mechanism of action and pharmacological activities of COL-3 as a potent inhibitor of several matrix metalloproteinases (MMPs), including MMP-1, MMP-2, MMP-8, MMP-9 and MMP-13 [34]. In fact, based on this latter ability of COL-3, clinical trials (phase I and II) have been performed to evaluate the treatment efficacy of the compound in patients with HIV-related Kaposi’s sarcoma, where MMPs play a pivotal role in the pathogenesis of the disease [35,36]. The results of these trials demonstrated that COL-3 is well tolerated, and that the response rate is about 41% in the recruited patients [36]. More importantly, treatment with COL-3 resulted in significant reductions in the plasma levels of MMP-2 and MMP-9 [36]. Interestingly, it has been shown that MMP-9 is also highly expressed in a fraction of CML patients during the blast crisis [37]. Upon considering these scientific observations and our findings in the present study, we suggest that COL-3 may be considered as a treatment modality for CML patients in blast crisis, where it not only inhibits the production of MMP-9, but also induces cell death in the leukemic cells. Indeed, the finding that COL-3 is well tolerated in patients with HIV-related Kaposi’s sarcoma provides an opportunity to evaluate the efficacy of this drug in CML patients in blast crisis.

## Supplementary Materials

The following are available online at https://www.mdpi.com/article/10.3390/jpm12010042/s1, Supplementary Materials and Methods, Figure S1: The effect of COL-3 on the viability of a mouse leukemic cell line C1498. Figure S2: The loss of mitochondrial membrane potential (ΔΨm) in COL-3-induced cell death. Figure S3: Immunocytoochemistry of K562 cells treated with COL-3. Figure S4: Caspase activation in doxycycline (Doxy), minocycline (Mino), COL-3 treated K562 cells. Figure S5: The effect of Z-VAD-FMK and necrostatin-1 on COL-3-induced K562 cell death. Figure S6: Intracellular ROS levels was measured by OxiSelectTM Intracellular ROS assay Kit. Figure S7: Effect of extracellular ions concentrations in COL-3 induced toxicity. Table S1: Characteristics of COL-3 induced cell death in K562 leukemic cells.
