# Supplementary material for: COL-3-Induced Molecular and Ultrastructural Alterations in K562 Cells
Source: J Pers Med. 2022 Jan 4;12(1):42. doi: 10.3390/jpm12010042 (PMC8778770; doi:10.3390/jpm12010042)
Supplement: Supplementary file 1 [file jpm-12-00042-s001.zip › jpm-1517243-supplementary - update.pdf]

Supplementary Material

COL-3-induced molecular and ultrastructural alterations in K562 cells

Mona Fares[1,2], Sandra Oerther[1,2], Kjell Hultenby[3], Danica Gubrianska[1,2]; Ying Zhao[1,2], Manuchehr Abedi-Valugerdi[1,2], and Moustapha Hassan[1,2*]

[1]Experimental Cancer Medicine, Division of Bio-molecular and Cellular Medicine (BCM), Novum, Karolinska Institutet, Stockholm, Sweden.

[2]Clinical Research Centre and Centre for Allogeneic Stem cell Transplantation, Karolinska University Hospital Huddinge, Sweden

[3]Department of Laboratory Medicine, Karolinska Institutet and Karolinska University Hospital, Stockholm, Sweden

Email: mona.fares@sll.se, sandra.oerther@ki.se, Kjell.Hultenby@ki.se, ying.zhao.1@ki.se, manuchehr.abedi-valugerdi@ki.se, moustapha.hassan@ki.se

*Corresponding author:
Moustapha Hassan
Experimental Cancer Medicine, Bio-molecular and Cellular Medicine (BCM), Novum, Department of Laboratory Medicine, Karolinska University Hospital Huddinge, 14186, Stockholm, Sweden.
E-mail: moustapha.hassan@ki.se

Running title: Mechanism of COL-3 toxicity in K562 cells

Key words: K562 cells, paraptosis, programmed necrosis, COL-3, m-calpain.

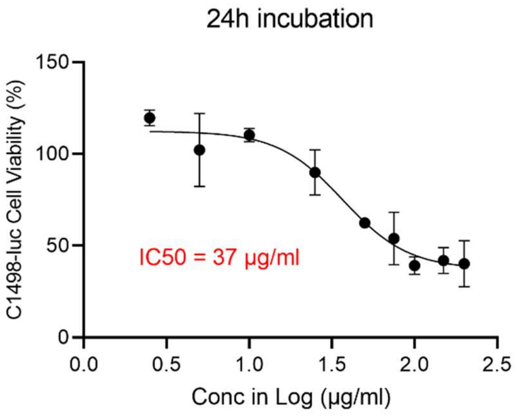

Figure S1: The effect of COL-3 on the viability of a mouse leukemic cell line C1498. Cell viability was studied using resazurin fluorescence WST-1 assay and expressed as percent of the control. Results are expressed as mean ± SD.

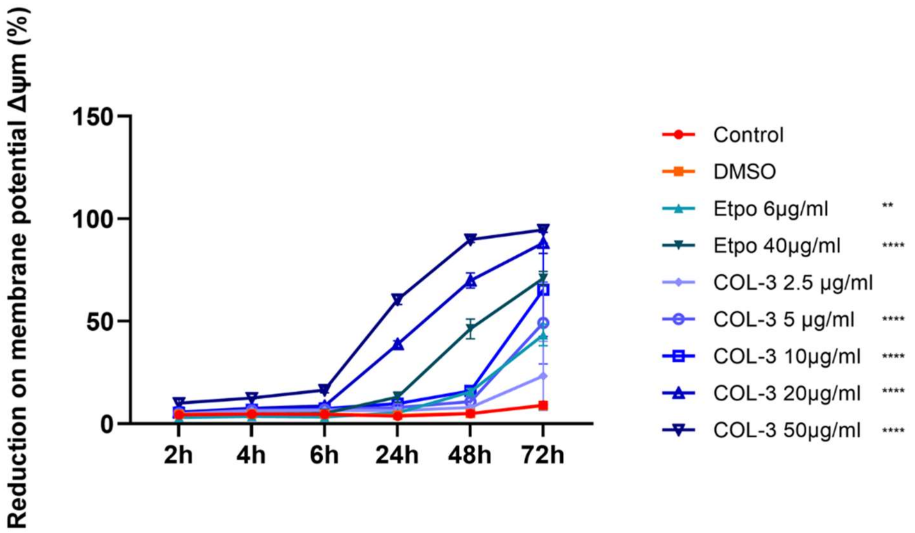

Figure S2: The loss of mitochondrial membrane potential (ΔΨm) in COL-3-induced cell death. Mitochondrial membrane potential was assessed using tetramethylrhodamine methyl ester (TMRM) and flow cytometry. K562 cells were incubated with COL-3 in final concentrations of 2.5, 5, 10, 20 and 50 µg/ml for 2, 4, 6, 24, 48 and 72 h. Cells incubated in DMSO in a final concentration of 0.2% were used as controls for solvent toxicity and cells incubated in complete medium served as controls. Results are presented as mean ± SD of three independent experiments.

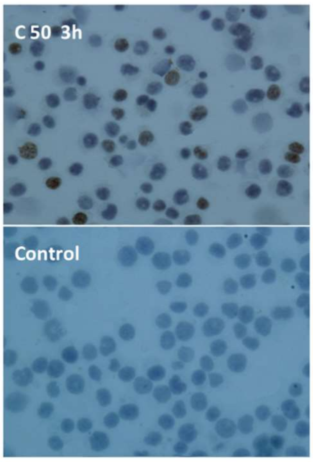

Figure S3: Immunocytoochemistry of K562 cells treated with COL-3 at the concentrations of 50 μg/ml for 3 h. Cells incubated in complete medium are used as a control. Double strand breaks were detected with γH2Ax staining. C = COL-3.

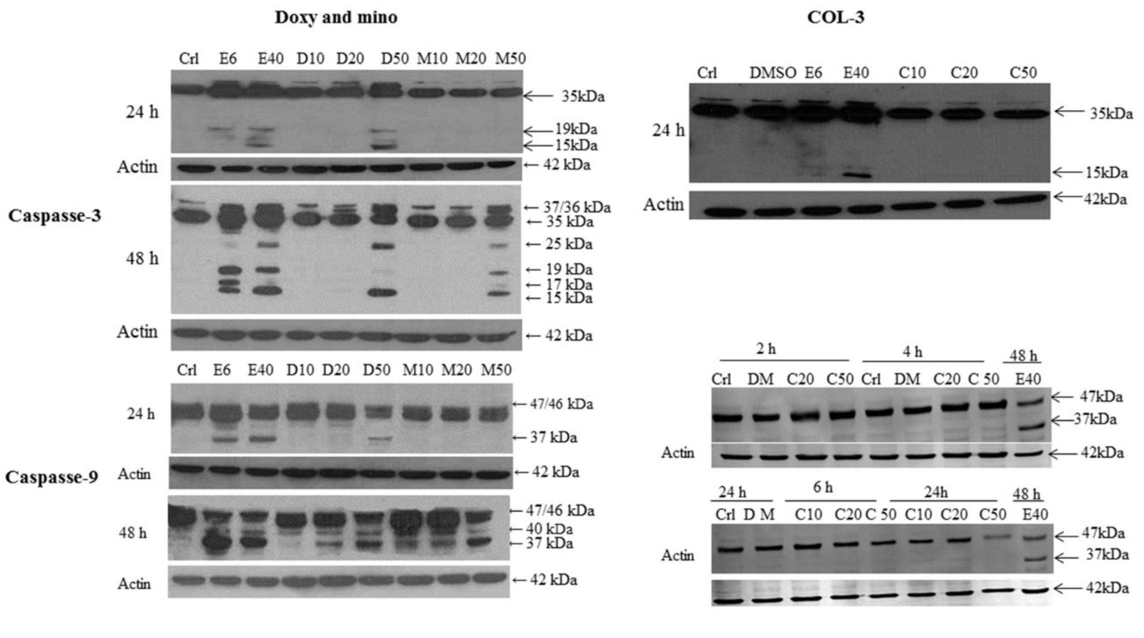

Figure S4: Caspase activation in doxycycline (Doxy), minocycline (Mino), COL-3 treated K562 cells. Cells were incubated with Doxy / Mino /COL-3 in concentrations of 10, 20 and 50 μg/ml. Cells treated with etoposide in final concentrations of 6 or 40 μg/ml served as controls for cell death and cells incubated in complete medium were used as controls. Specific protein bands were determined using PAGE and WB. β-actin was used a control of protein loading. Crl: control; D: doxycycline; M: minocycline; E: etoposide; C: COL-3; DM: DMSO.

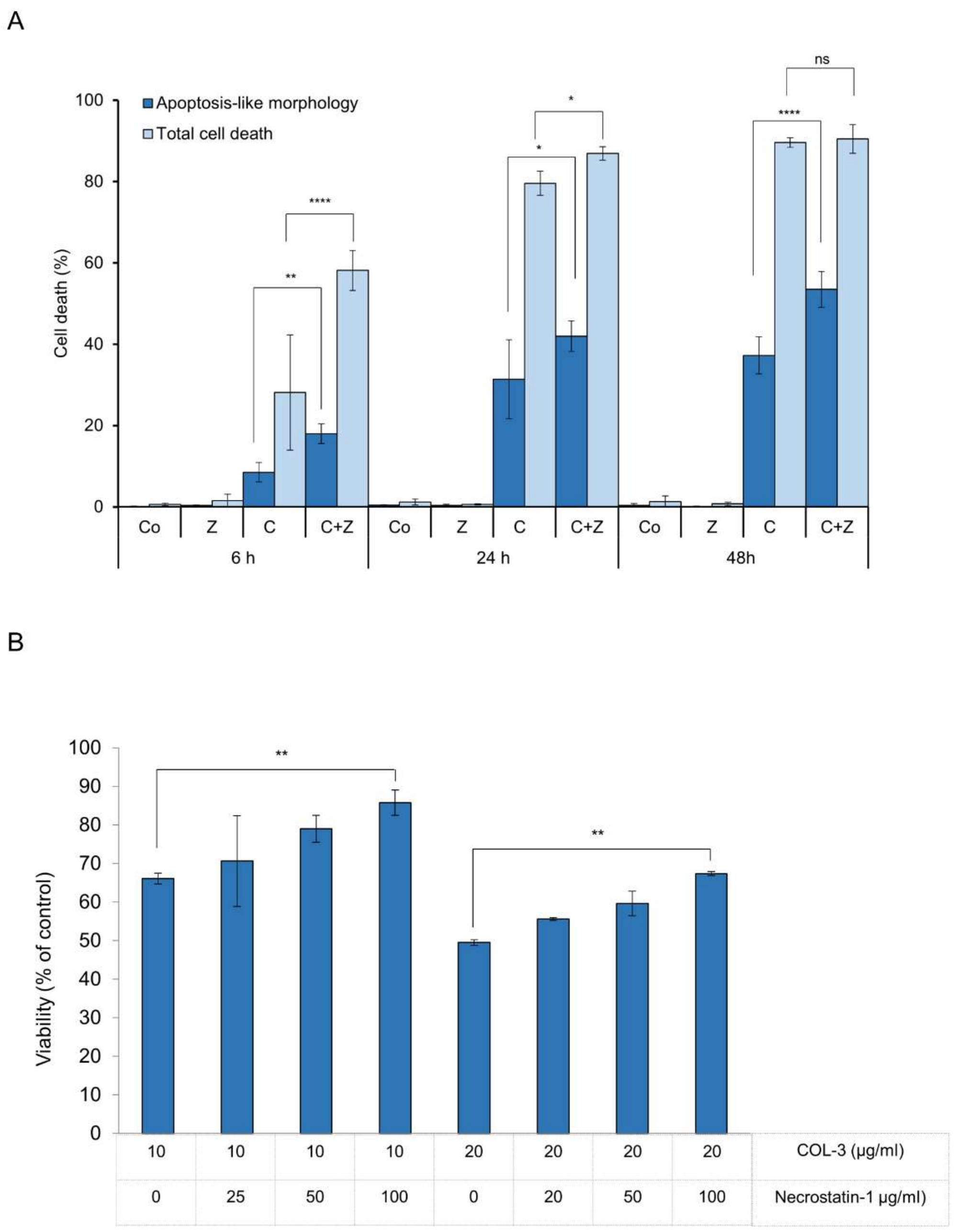

Figure S5: The effect of Z-VAD-FMK and necrostatin-1 on COL-3-induced K562 cell death. (A) K562 cells were incubated with Z-VAD-fmk with a final concentration of 100 µM for 1 h before treatment with COL-3 in a final concentration of 50 µg/ml for 6, 24 and 48 h. Cells incubated in 0.1% DMSO served as controls for solvent toxicity, cells incubated in complete medium as controls. Cell morphology was assessed on cytospun MGG stained slides. A minimum of 400 cells was counted per slide. (B). K562 cells were incubated with necrostatin-1 with a final concentration of 25, 50 and 100 µM for 1 h before treatment with COL-3 in a final concentration of 10 and 20 µg/ml for 24 h. Cells incubated in complete medium are used as a control. Cell viability was measured by MTT assay. Results are presented as mean ± SD

of three independent experiments. Co = control; C = COL-3; Z = Z-VAD-fmk. *: P<0.05; **: P<0.01; ****: P<0.0001, and ns: not significant.

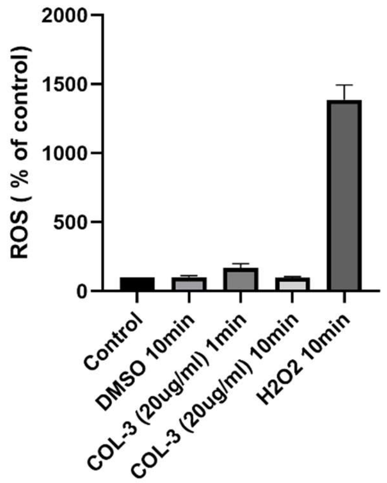

Figure S6: Intracellular ROS levels was measured by OxiSelectTM Intracellular ROS assay Kit. Cells were incubated with 100 μM of 2'-7'-dichlorodihydrofluorescin (DCFH-DA) in media for 1 h at 37 °C, then treated with COL-3 at a final concentration of 20 μg/ml for 1 and 10 min. DCF fluorescence intensity was measured at 485/520 nm wavelengths using FLUOstar Optima. Intracellular ROS levels showed no change following treatment with COL-3 (20 μg/ml). H_2O_2 (100 mM) was used as a positive control.

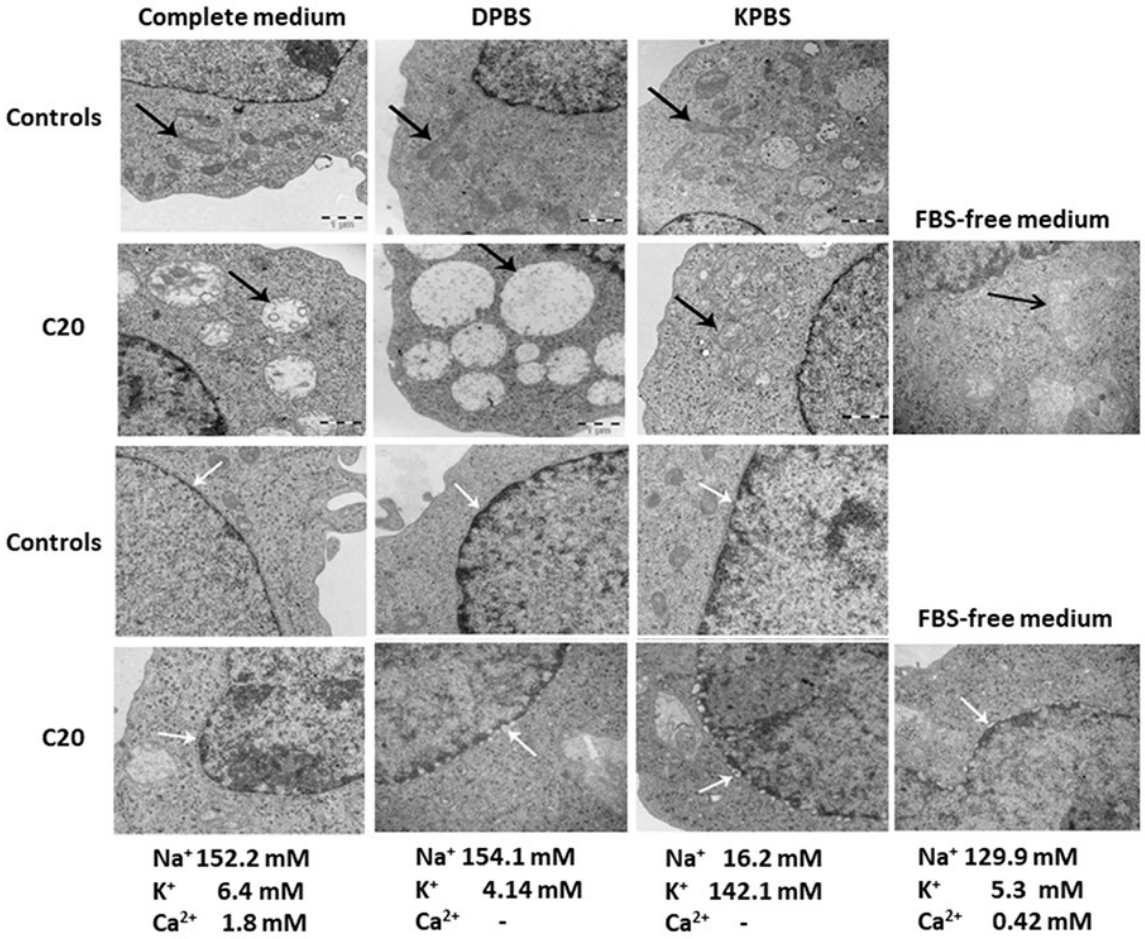

Figure S7. Effect of extracellular ions concentrations in COL-3 induced toxicity

Cells were treated with COL-3 in a final concentration of 20 µg/ml for 10 min in four different solutions: complete medium, Dulbecco's PBS (DPBS), potassium-rich PBS (KPBS) and FBS-free medium. Ultrathin sections were prepared in glutaraldehyde-fixed and osmium tetroxide-postfixed cells and analysed with TEM. The mitochondria (black arrow) and the nuclear envelop (white arrow) are presented.

Supplementary Table 1: ***Characteristics of COL-3 induced cell death in K562 leukemic cells***

Observable parameter	*Apoptosis*	*Necrosis*	*Paraptosis*	*Programmed necrosis*	*COL-3 20 µg/ml*	*COL-3 5 µg/ml*
Nuclear Fragmentation	+	-	-	-	-	-
Chromatin condensation	+	-	-	+	+	+
Apoptotic bodies	+	-	-	-	-	-
Annexin V (early)	+	-	-	+	+	-
Propedium iodide(early)	-	+	- Late +ve?	+	+	-
Cytoplasmic vaculation	-	+	+	-	-	-
Mitochondrial swelling	+ (sometimes)	+	+ (Late)	+ Late??	+	+
ER swelling	-	-	+	-	+	+
DNA fragmentation	+ (*internucleos-omal*)	+ (diffuse)	-	+ HMW	Nuclear tAIF (+)	tAIF
Caspase-3 activation	+	-	-	-	-	-
Caspase-9 activation	+ (Apaf-1 dependent)	-	+ (Apaf-1 independent)	-	+? Zymogen No Apaf-1 assembly	+? Zymogen No Apaf-1 assembly
AIF processing	+ (sometimes)	-	-	+	+	+
PARP cleavage	+ (85kDa fragment)	+ (50-62 kDa fragments)	-	-	-	-

Inhibition by zVAD.fmk	+	-	-	-	-	
Bcl-xL inactivation	+	-	-	+	-	-
Calpain activation	±	-	±	+	+	+
